# Boron Derivatives Accelerate Biofilm Formation of Recombinant *Escherichia coli* via Increasing Quorum Sensing System Autoinducer-2 Activity

**DOI:** 10.3390/ijms23158059

**Published:** 2022-07-22

**Authors:** Huan Chen, Cheng-Hai Yan, Yu-Fan Zhan, Li-Tian Geng, Lin-Lin Zhu, Lu-Chan Gong, Jun Wang

**Affiliations:** 1 Jiangsu Key Laboratory of Sericultural Biology and Biotechnology, School of Biotechnology, Jiangsu University of Science and Technology, Zhenjiang 212100, China; 192310002@stu.just.edu.cn (H.C.); biojustych@163.com (C.-H.Y.); 211111901120@stu.just.edu.cn (Y.-F.Z.); genglt_28@163.com (L.-T.G.); zhulinlin1208@163.com (L.-L.Z.); lcgong@just.edu.cn (L.-C.G.); 2 Key Laboratory of Silkworm and Mulberry Genetic Improvement, Ministry of Agriculture and Rural Affairs, Sericultural Research Institute, Chinese Academy of Agricultural Sciences, Zhenjiang 212100, China

**Keywords:** quorum sensing, autoinducer-2, boron, biofilm, extracellular polymeric substances

## Abstract

Boron is an essential element for autoinducer-2 (AI-2) synthesis of quorum sensing (QS) system, which affects bacterial collective behavior. As a living biocatalyst, biofilms can stably catalyze the activity of intracellular enzymes. However, it is unclear how boron affects biofilm formation in *E. coli*, particularly recombinant *E. coli* with intracellular enzymes. This study screened different boron derivatives to explore their effect on biofilm formation. The stress response of biofilm formation to boron was illuminated by analyzing AI-2 activity, extracellular polymeric substances (EPS) composition, gene expression levels, etc. Results showed that boron derivatives promote AI-2 activity in QS system. After treatment with H_3_BO_3_ (0.6 mM), the AI-2 activity increased by 65.99%, while boron derivatives increased the biomass biofilms in the order H_3_BO_3_ > NaBO_2_ > Na_2_B_4_O_7_ > NaBO_3_. Moreover, treatment with H_3_BO_3_ (0.6 mM) increased biomass by 88.54%. Meanwhile, AI-2 activity had a linear correlation with polysaccharides and protein of EPS at 0–0.6 mM H_3_BO_3_ and NaBO_2_ (R^2^ > 0.8). Furthermore, H_3_BO_3_ upregulated the expression levels of biofilm formation genes, quorum sensing genes, and flagellar movement genes. These findings demonstrated that boron promoted biofilm formation by upregulating the expression levels of biofilm-related genes, improving the QS system AI-2 activity, and increasing EPS secretion in *E. coli*.

## 1. Introduction

Biofilm is composed of bacteria and extracellular polymeric substances (EPS) secreted by bacteria, which are the primary means by which microorganisms survive in the natural environment [1]. EPS is the main component of biofilms and provides mechanical stability. Biofilms separate bacteria from the external environment and provide a stable environment [2], acting as a living catalyst with great advantages in the field of biological resource transformation [3]. Biofilm has been widely used in industrial fields, such as bioremediation [4], wastewater treatment [5], fine chemical production [6], fermentation, and biotransformation [7]. However, bacterial biofilms grow disorderly and loosely, resulting in limited mass transfer and reduced reactor efficiency [8]. To improve the reproduction ability of engineering bacteria biofilm in industrial production, it is urgent to find a method to regulate the formation of biofilms.

Bacterial collective behavior can be coordinated by a process termed quorum sensing (QS) [9]. In this process, the chemical signaling molecule autoinducer (AI) secreted by bacteria accumulates with increasing bacterial density. When the AI level reaches a certain threshold, gene expression in a bacterium is activated, causing the population to produce a coordinated phenotypic response. Quorum sensing plays an important regulatory role in biofilm formation, bacterial persistence, and so on [10]. For instance, Xuan et al. found that QS upregulated the expression of lipopolysaccharide synthesis and promoted biofilm development in *P. aeruginosa* [11]. Tsao et al. re-engineered the native QS regulon, in which *Escherichia coli* secreted QS signal autoinducer-2 (AI-2) initiated and guided the high-level expression of recombinant proteins [12]. Therefore, regulating the QS system of engineering bacteria is an effective way to improve the adhesion and growth of biofilms in industrial production.

Further, previous studies showed that the direct addition of exogenous QS signal molecules could also regulate bacterial collective behavior [13]. Xiong et al. reported that AI-2 played an important role in the maturation and maintenance integrity morphological in aerobic bacteria [14]. Ren et al. found that the signal molecules released by aerobic bacteria could stimulate the adsorption, aggregation, and growth of floating cells, which was conducive to the regeneration of biofilm [15]. Since AI-2 is not easy to be artificially synthesized, it is expensive to directly add exogenous AI-2 to regulate the QS system. Boron is an essential element for synthesizing QS system AI-2, and the active AI-2 is produced by adding naturally occurring borate to the AI-2 precursor [16,17]. Semmelhack et al. found that affinity columns with borate resin were effective in providing and purifying AI-2 of *V. harveyi* from the biosynthetic product [18]. Zhang et al. revealed that adding boron to the sequencing batch reactor (SBR) can increase the activity of AI-2 and promote the secretion of EPS [19]. At present, there are few studies on the role of boron derivatives in the QS system, and the regulatory mechanism of boron derivatives on *E. coli* biofilm formation is unclear, particularly for recombinant *E. coli* containing the intercellular catalytic enzyme. As a result, it is critical to investigate the effect of boron derivatives on AI-2 activity and biofilm formation.

In this study, the recombinant *E. coli* containing the intercellular catalytic enzyme is the research object. The effects of boron derivatives on the growth curve and the formation of biofilms were determined, and the changes in the content of EPS, the main components of biofilms were analyzed. On this basis, through real-time quantitative polymerase chain reaction (RT-qPCR) experiments, the effect of boron on the transcription level of genes related to biofilm formation was analyzed, and the surface of biofilms treated with boron derivatives was characterized. Finally, the effect of boron on the enzyme catalysis of recombinant *E. coli* was explored by biofilm formation. These findings help clarify the relationship between boron derivatives in the environment, and biofilm formation.

## 2. Results and Discussion

### 2.1. Effects of Boron Derivatives on the AI-2 Activity of Recombinant E. coli

**Figure 1:**
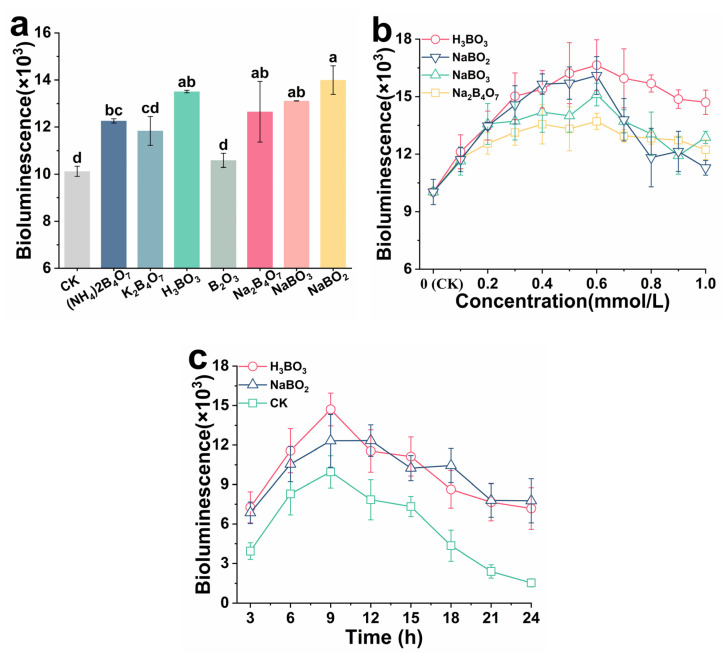
Effects of boron derivative types (**a**), concentration (**b**), and cultivation time (**c**) on the AI-2 activity of *E. coli*. Culture conditions: (**a**) the concentration of boron derivatives was 0.25 mM and the incubation time was 6 h; (**b**) boron derivatives with different concentrations of H_3_BO_3_, NaBO_2_, NaBO_3,_ and Na_2_B_4_O_7_ were added, respectively, and the culture time was 6 h; (**c**) the concentration of boron derivatives was 0.6 mM. AI-2 activity was detected in the sterile supernatant every 3 h and continuously monitored for 24 h. The different letters above the bars indicate significant differences (*p* < 0.05).

The formation of biofilm in *E. coli* is controlled by AI-2. To clarify the effect of boron on biofilm formation, the activity of AI-2 was detected. As shown in Figure 1a, the boron derivatives had different effects on AI-2 activity at the same concentration (0.25 mM). Compared to the control group, AI-2 activity significantly improved after adding boron derivatives (*p* < 0.05), except for B_2_O_3_. After adding H_3_BO_3_, NaBO_2_, NaBO_3_, Na_2_B_4_O_7_, K_2_B_4_O_7_, and (NH_4_)_2_B_4_O_7_, AI-2 activity increased by 33.44%, 38.28%, 29.58%, 25.01%, 16.95%, and 21.16%, respectively. AI-2 was derived from precursor 4,5-dihydroxy-2,3-pentanedione (DPD) [20]. DPD was reported to be quite unstable toward rearrangement and oligomerization, but it can exist as a balanced mixture of borate complexes and generate active AI-2 [21]. The findings indicate that H_3_BO_3_ is not the only factor that promotes AI-2 activity in recombinant *E. coli*, however, most boron derivatives, particularly H_3_BO_3_, NaBO_2_, NaBO_3_, and Na_2_B_4_O_7_, could be used.

To explain the effect of boron derivative concentration on AI-2 activity, the concentration was gradually increased from 0 to 0.6 mM using H_3_BO_3_, NaBO_2_, NaBO_3_, and Na_2_B_4_O_7_. Compared with no boron derivative addition, after 0.6 mM treatment, H_3_BO_3_ increased the AI-2 activity by 65.99%, NaBO_2_ increased by 60.58%, NaBO_3_ increased by 50.71%, and Na_2_B_4_O_7_ increased by 36.71% (Figure 1b). When the concentration was higher than 0.6 mM, the promoting effect of boron derivatives on AI-2 activity was weakened. Eventually, H_3_BO_3_ increased by 46.66%, NaBO_2_ increased by 12.57%, NaBO_3_ increased by 21.98%, and Na_2_B_4_O_7_ increased by 28.41% when treated with boron derivatives of 1.0 mM. There was a dose-dependent relationship between boron derivatives and AI-2 activity at low concentrations (0–0.6 mM). AI-2 activity was increased when low concentrations of boron derivatives were added, indicating that *E. coli* could sense boron derivatives. The promotion of AI-2 activity was impeded by the high concentration of boron derivatives. Boron is biologically essential, and while it is necessary for cell nutrition, an excess of it can be toxic [22]. Therefore, 0.6 mM is the optimal concentration of boron derivatives in recombinant *E. coli* to induce AI-2 activity, especially for H_3_BO_3_ and NaBO_2_. The AI-2 activity with cultivation time was monitored using H_3_BO_3_ and NaBO_2_ for 24 h.

As shown in Figure 1c, AI-2 activity rapidly increased from 0 h to9 h, and then gradually decreased. AI-2 activity peaked at 9 h, increasing by 47.62% in H_3_BO_3_ and by 23.71% in NaBO_2_ compared to the control group. When cultured for 24 h, AI-2 activity was almost undetectable in the control group, while the AI-2 activity could still be detected in the treatment groups containing H_3_BO_3_ and NaBO_2_. AI-2 activity increased till the late exponential phase and then diminished slowly in the stationary phase [23]. A possible reason was that the signal molecule AI-2 was re-absorbed by the bacteria or attached to their cell membrane, which regulated some essential physiological functions [24]. The boron derivatives not only increased the activity of AI-2 in *E. coli*, but also prolonged the existing time of activity. Thus, the boron derivatives as exogenous additives could effectively regulate QS system by AI-2.

### 2.2. Growth Analysis of Biofilm after the Adding of Boron Derivatives

**Figure 2:**
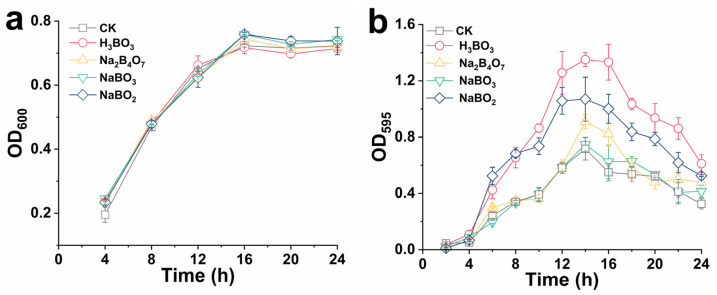
Effects of boron derivatives types on the growth curve (**a**) and *E. coli* biofilm formation (**b**). Culture conditions: (**a**) the concentration of boron derivatives was 0.6 mM, the culture temperature was 37 °C, and the rotation speed was 220 rpm; (**a**) the concentration of boron derivatives was 0.6 mM, the culture temperature was 37 °C and the biofilm was cultured in a 24-well plate.

To verify the effect of boron derivative on biofilm formation, the growth curve of recombinant *E. coli* was detected (Figure 2a). This addition had no effect on the strain’s growth, which had reached a stable phase after 15 h. Therefore, the period of the highest biofilm index of recombinant *E. coli* was at the initial stage of stable growth of the strain, which corresponded to the period with the highest AI-2 activity in previous studies [25]. Figure 2b illustrates the growth of recombinant *E. coli* biofilm with H_3_BO_3_, NaBO_2_, and NaBO_3_. The total biomass and growth curve of recombinant *E. coli* were monitored for 24 h. As the culture time increased, the total biomass of recombinant *E. coli* gradually increased, reaching the highest at 14 h. Compared to the control group, after adding H_3_BO_3_, NaBO_2_, and Na_2_B_4_O_7_, the biomass increased by 88.54%, 49.17%, and 27.13%, respectively. There was no difference between the total biomass of NaBO_3_ and the control group. However, the biomass of all groups continued to decrease after 14 h, the reason may be that the biofilm growth of recombinant *E. coli* had entered the spreading period [26]. Boron is the primary component of AI-2, an intercellular communication signal molecule [27]. Huang et al. found that exogenous addition of AI-2 synthesized in vitro regulated biofilm formation and cell adhesion [28]. In this study, boron derivatives also increased AI-2 activity. Therefore, it was preliminarily speculated that boron derivatives increased biofilm biomass by regulating AI-2 activity in *E. coli*.

### 2.3. Biofilm Assay by SEM and CLSM

**Figure 3:**
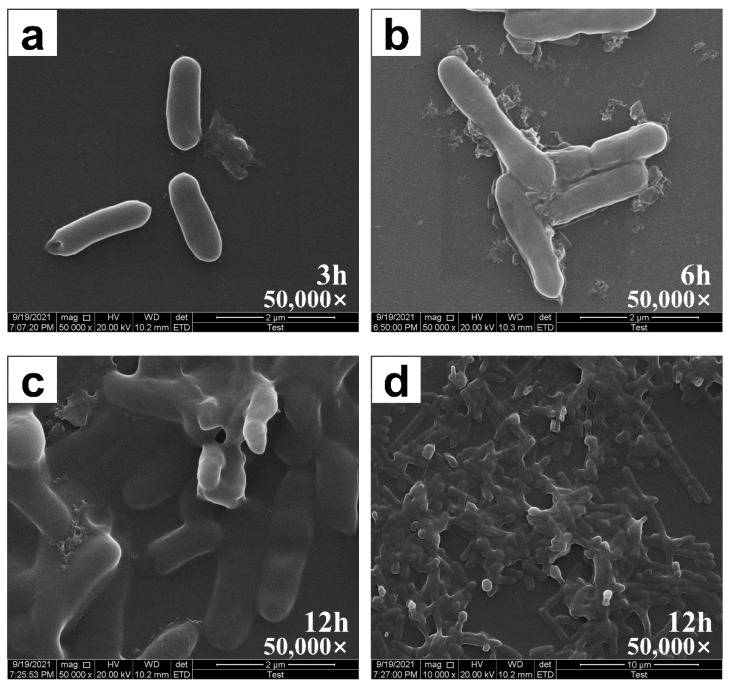
SEM diagrams of *E. coli* biofilm formation. (**a**) Biofilms cultured for 3 h (50,000×); (**b**) biofilms cultured for 6 h (50,000×); (**c**) biofilms cultured for 12 h (50,000×); (**d**) mature biofilms from a large perspective (10,000×). The incubation time was 12 h and no boron derivatives during this cultivation.

The biofilm formation was detected by SEM, indicating the formation of recombinant *E. coli* biofilm on glass slides. Bacteria adhere to various surfaces and secrete various highly organized, coordinated, and functional extracellular substances to form biofilms [29]. Figure 3a shows that when *E. coli* was cultured for 3 h, the cell was only adsorbed on the glass surface. When the strain was cultured for 6 h (Figure 3b), a small number of EPS was observed around the bacteria, which improved *E. coli* adhesion by bringing together a large number of EPS-wrapped bacteria to form mature biofilms with good network structures (Figure 3c,d). The formation of EPS separates bacteria from the external environment, providing a stable environment for bacteria to grow and metabolize [30]. Recombinant *E. coli* can secrete EPS and form mature biofilm within 12 h, indicating that the strain has a strong biofilm forming ability and simple culture conditions.

**Figure 4:**
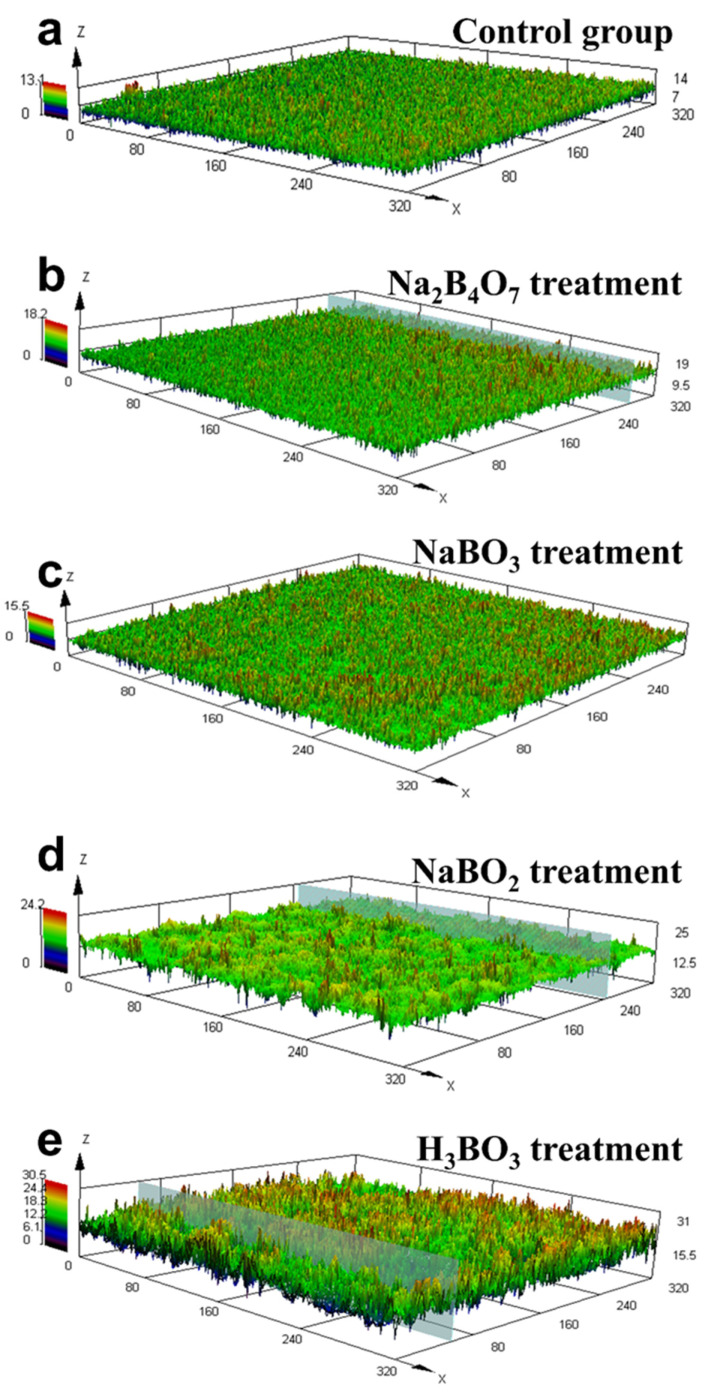
CLSM diagrams of biofilms cultured with no boron derivatives (**a**), Na_2_B_4_O_7_ (**b**), NaBO_3_ (**c**), NaBO_2_ (**d**), and H_3_BO_3_ (**e**) treatment. The concentration of all boron derivatives was 0.6 mM, and the incubation time was 12 h. X and Y axes represent the length of the 3D map of the biofilm, and Z-axis is the thickness of biofilms (μm).

Figure 4 illustrates the CLSM images of biofilms with varying boron derivatives, while Table 1 shows the average thickness of the biofilms in corresponding CLSM diagrams. Figure 4a shows that a thin and uniform biofilm was formed in the control group without boron derivatives after culturing for 12 h. Table 1 determines that the average thickness of the biofilm in the control group was 4.80 μm. The biofilm thicknesses were 5.63 μm and 5.66 μm after adding NaBO_3_ and Na_2_B_4_O_7_, respectively, which were significantly different from those of the control group. The biofilm thicknesses were 8.11 μm and 11.13 μm after adding NaBO_2_ and H_3_BO_3_, respectively, which had significant differences compared to the control group (*p* < 0.05). Figure 4 visually shows the same results as in the table. Thick and rough biofilms were observed after adding NaBO_2_ and H_3_BO_3_. Notably, potassium ions in buffer salt increased the expression of the QS gene *luxS* and promoted biofilm formation of *Lactobacillus plantarum* [31]. This study found that boron derivatives had the same promoting effect on *E. coli* biofilm, and NaBO_2_ and H_3_BO_3_ were determined as the most effective substance.

### 2.4. Effect of Adding Boron Derivatives on the EPS of Recombinant E. coli

**Figure 5:**
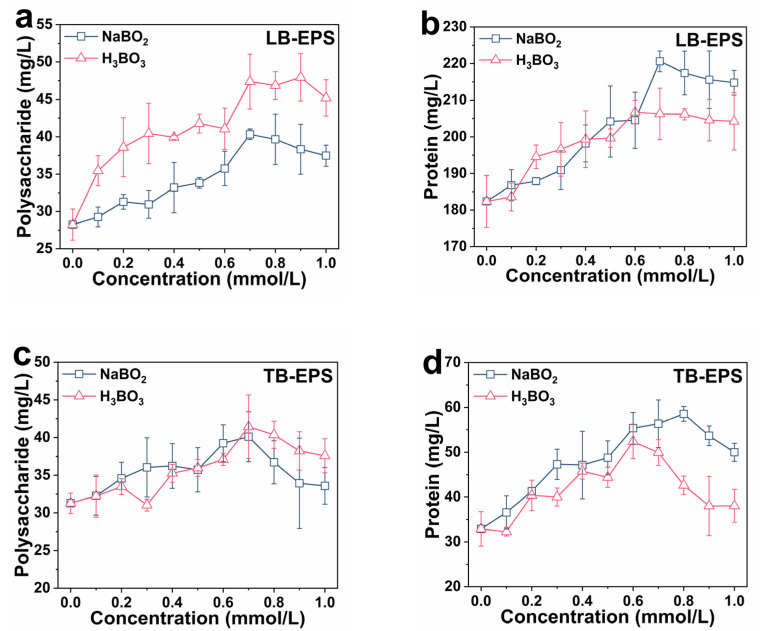
Effects of boron derivatives concentration on LB-EPS-polysaccharide (**a**), LB-EPS-protein (**b**), TB-EPS-polysaccharide (**c**), and TB-EPS-protein (**d**). Extracting conditions: The bacterial liquid was centrifuged and collected. The bacterial sludge was suspended at 70 °C and 0.05% NaCl. Immediately, the mixture was centrifuged and the supernatant was loosely bound EPS (LB-EPS). Sludge deposition in LB-EPS was suspended with 0.05% NaCl. The mixture was placed in a water bath at 70 °C for 30 min, centrifuged and the supernatant was taken as tightly bound EPS (TB-EPS).

Figure 5a,b illustrates the effect of boron derivative concentration on LB-EPS. The addition of boron derivatives effectively increased LB-EPS content in the bacteria sludge. As H_3_BO_3_ and NaBO_2_ concentrations increased, polysaccharide and protein contents gradually rose and tended to level off. When boron derivatives content was 0 mmol/L, the LB-EPS polysaccharide and protein concentrations were 28.23 mg/L and 182.33 mg/L, respectively. When H_3_BO_3_ concentration was 0.7 mM, LB-EPS content was the highest. The LB-EPS polysaccharide and protein concentrations increased by 67.80% (47.23 mg/L) and 13.12% (206.25 mg/L), respectively. When NaBO_2_ concentration was 0.7 mM, LB-EPS content was also the highest. The LB-EPS polysaccharide and protein concentrations increased by 42.83% (40.32 mg/L) and 20.99% (220.61 mg/L), respectively.

Figure 5c,d depicts the effect of boron derivative concentration on TB-EPS. Adding boron derivatives also effectively increased the TB-EPS content in the bacteria sludge. The polysaccharide and protein contents initially increased as H_3_BO_3_ and NaBO_2_ concentrations increased before falling. TB-EPS polysaccharide and protein concentrations were 31.27 mg/L and 32.90 mg/L, respectively, without boron derivatives added. When H_3_BO_3_ concentration was 0.7 mM, the TB-EPS polysaccharide and protein concentrations increased by 27.39% (40.11 mg/L) and 71.13% (56.35 mg/L), respectively. When NaBO_2_ concentration was 0.7 mM, the TB-EPS polysaccharide and protein concentrations increased by 32.52% (41.44 mg/L) and 51.17% (49.92 mg/L), respectively.

EPS are complex mixtures composed of polysaccharides, proteins, and some other compounds, which can form a highly hydrated gel matrix [32]. EPS displayed a dynamic double-layered structure of LB-EPS and TB-EPS. Huang et al. found that the exogenous addition of QS signal N-acyl-homoserine lactones (AHL) increased the content of LB-EPS, but did not change the composition of TB-EPS [33]. Compared with LB-EPS, the structure of TB-EPS was tighter in the formation process [34]. However, in this study, the addition of boron derivatives increased not only the content of LB-EPS but also tight TB-EPS in *E. coli* biofilm. When added at a suitable concentration, boron derivatives can significantly promote the growth of biofilm. Considering that boron derivatives are inexpensive and abundant, they are excellent additives for promoting biofilm formation.

### 2.5. Linear Correlation between the Activity of Signal Molecule AI-2 and EPS

**Figure 6:**
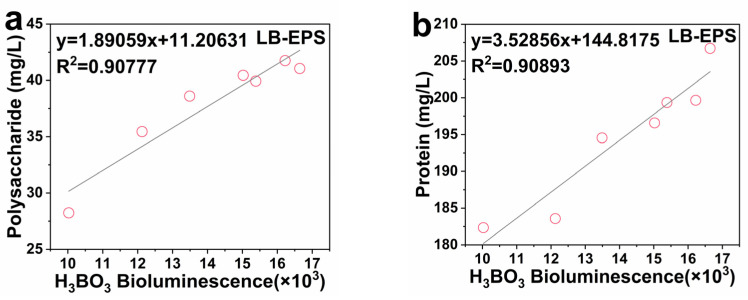

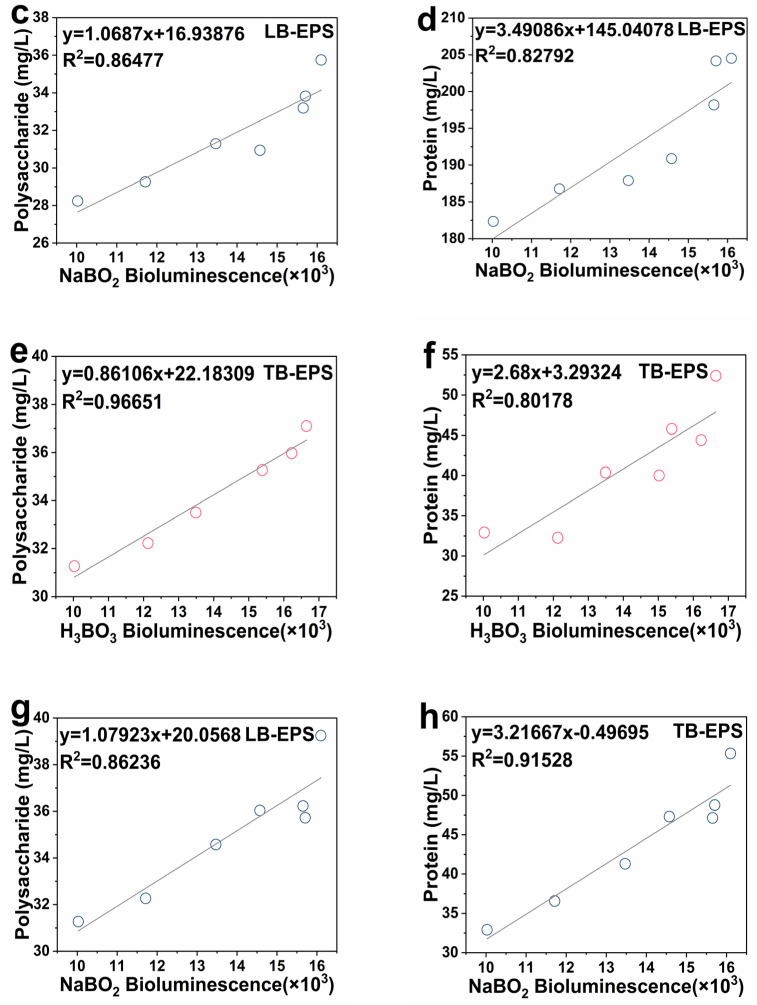
Linear correlation analysis of EPS and AI-2 activity in *E. coli*, (**a**) AI-2 activity and LB-EPS-polysaccharide under H_3_BO_3_ treatment, (**b**) AI-2 activity and LB-EPS-protein under H_3_BO_3_ treatment, (**c**) AI-2 activity and LB-EPS-polysaccharide under NaBO_2_ treatment, (**d**) AI-2 activity and LB-EPS-protein under NaBO_2_ treatment, (**e**) AI-2 activity and TB-EPS-polysaccharide under H_3_BO_3_ treatment, (**f**) AI-2 activity and TB-EPS-protein under H_3_BO_3_ treatment, (**g**) AI-2 activity and TB-EPS-polysaccharide under NaBO_2_ treatment, and (**h**) AI-2 activity and TB-EPS-protein under NaBO_2_ treatment.

Boron has the potential to promote AI-2 and increase EPS; however, it is unclear whether there is a linear relationship between AI-2 and EPS. Figure 6a–d illustrate the linear correlation analysis of LB-EPS and AI-2 activity. After adding H_3_BO_3_, AI-2 activity and polysaccharides, and AI-2 activity and proteins had a good linear correlation in LB-EPS. The correlation between AI-2 activity and LB-EPS-polysaccharides was R^2^ = 0.90777, while that between AI-2 activity and LB-EPS-proteins was R^2^ = 0.90893. After adding NaBO_2_, AI-2 activity and polysaccharides, and AI-2 activity and proteins, also had a good linear correlation in LB-EPS. The correlation between AI-2 activity and LB-EPS-polysaccharides was R^2^ = 0.86477, while that between AI-2 activity and LB-EPS-proteins was R^2^ = 0.82792.

Figure 6e–h depicts the linear correlation analysis of TB-EPS and AI-2 activity. Such activity treated with H_3_BO_3_ and NaBO_2_ had a good linear correlation with proteins and polysaccharides in TB-EPS. After adding H_3_BO_3_, the correlation between AI-2 activity and TB-EPS-polysaccharides was R^2^ = 0.96651, while that between AI-2 activity and TB-EPS-proteins was R^2^ = 0.80178. After adding NaBO_2_, the correlation between AI-2 activity and TB-EPS-polysaccharides was R^2^ = 0.86236, while that between AI-2 activity and LB-EPS-proteins was R^2^ = 0.91528. PN maintained the stability of internal structure and biomass retention of the biofilm based on high binding strength, and PS further improved the adhesion of microorganisms and maintained the stability of the external structure [35]. Nahm et al. found that there was a positive correlation between the QS signal AHL concentration and the amount of biofilm formed for *Aeromonas* sp. and *Leclercia* sp. [36]. Since the QS signal molecule, AI-2 activity had good correlations with LB-EPS and TB-EPS, it can be inferred that AI-2 was affected by the concentration of boron derivatives which regulate the secretion of EPS, and jointly promote the formation of biofilm in *E. coli*.

### 2.6. Transcription Level of Biofilm Formation, QS and Flagellar Movement Related Genes

**Figure 7:**
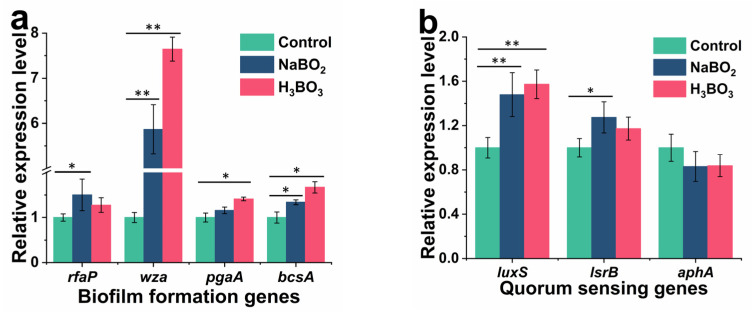

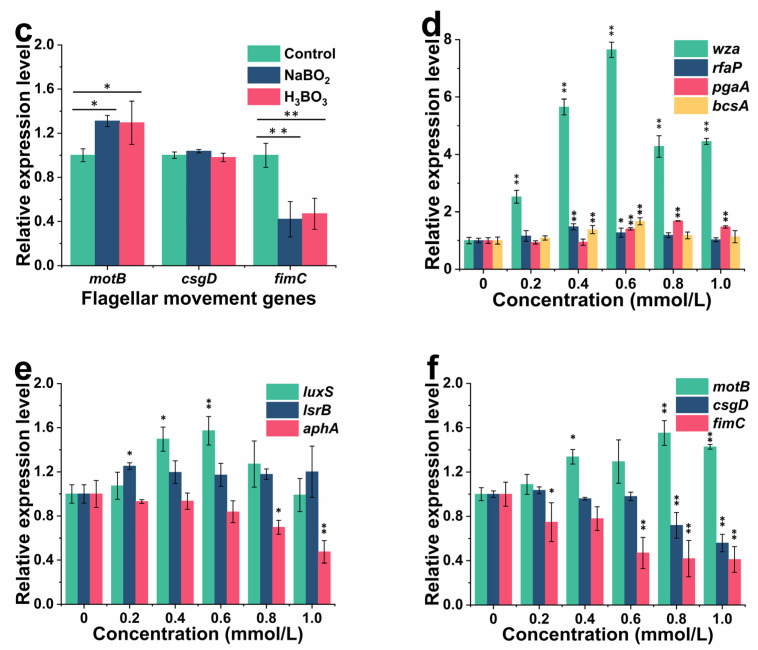
Effects of boron derivatives types on biofilm formation genes (**a**), quorum sensing genes (**b**), flagellar movement genes (**c**) expression levels, and effects of H_3_BO_3_ concentrations on biofilm formation genes (**d**), quorum sensing genes (**e**), and flagellar movement genes (**f**) expression levels. The expression of genes was analyzed by real-time PCR with three independent biological replicates. * *p* < 0.05: significant compared with the control, ** *p* < 0.01: highly significant compared with the control.

To explore how the boron affects biofilm formation, AI-2 and EPS, and the expression of related genes was detected. Figure 7a illustrates the effect of adding boron derivatives on the relative expression of *E. coli* biofilm formation genes (*rfaP, wza, pgaA,* and *bcsA*). After adding H_3_BO_3_, all genes were upregulated except *rfaP*. The expressions levels of *wza*, *pgaA*, and *bcsA* genes were upregulated by 7.64-fold, 1.41-fold, and 1.67-fold, respectively. After adding NaBO_2_, all genes were upregulated except *pgaA*. The *rfaA, wza*, and *bcsA* gene expression levels were upregulated by 1.50-fold, 5.87-fold, and 1.34-fold, respectively. Figure 7d depicts the effect of H_3_BO_3_ concentration on biofilm formation genes. The expression level of *wza* was significantly upregulated with 0.2–1.0 mM with H_3_BO_3_ treatment (*p* < 0.05). The promotion effect was best at a concentration of 0.6 mM, in which the relative expression of *wza* was upregulated by 7.5-fold. The expression level of *rfaP* significantly upregulated at medium concentrations (0.4 mM and 0.6 mM) with H_3_BO_3_ treatment (*p* < 0.05). The expression level of *pgaA* was significantly upregulated at concentrations from 0.6 mM to 1.0 mM with H_3_BO_3_ treatment (*p* < 0.05). The expression level of *bcsA* significantly upregulated at concentrations from 0.4 mM to 0.6 mM with H_3_BO_3_ treatment (*p* < 0.05). Furthermore, *rfaP, pgaA,* and *bcsA* were important genes for regulating *E. coli* biofilm synthesis [37]. The *rfaP* gene was responsible for the assembly of the lipopolysaccharide core [38]. The *pgaA* and *bcsA* genes are involved in the production and export of cellulose. Upregulation of biofilm formation genes indicated that the presence of boron derivatives induced biofilm formation gene expression in recombinant *E. coli*, which could also be a reason for increased EPS production during biofilm development when adding boron derivatives.

Figure 7b shows the effect of boron derivatives on *E. coli* QS genes (*luxS, lsrB,* and *aphA*). After adding H_3_BO_3,_ the *luxS* gene expression was upregulated by 1.57-fold, while after adding NaBO_2_, the *luxS* and *lsrB* gene expression was upregulated by 1.48-fold and 1.27-fold, respectively, except *aphA.* Figure 7e illustrates the effect of H_3_BO_3_ concentration on QS genes. The expression level of *luxS* was significantly upregulated at concentrations from 0.4 mM to 0.6 mM H_3_BO_3_ treatment (*p* < 0.05). The expression level of *lsrB* was significantly upregulated by 0.2 mM H_3_BO_3_ treatment (*p* < 0.05). The expression level of *aphA* was significantly downregulated at high concentrations from 0.8 mM to 1.0 mM H_3_BO_3_ treatment (*p* < 0.05). The *luxS* gene encodes S-ribosylhomocysteinase, an enzyme which generates the autoinducer-2 (AI-2) precursor of DPD from S-ribosyl-L -homocysteine [20]. When AI-2 enters cells, it firstly binds to the AI-2 transports system substrate-binding protein encoded by *lsrB*. *AphA* was a master regulator of QS at low cell density, and plays an essential role in the expression of genes associated with physiology and virulence in gram-negative pathogens [39]. Recombinant *E. coli* altered the genes to transition from low to high density environments, influencing biofilm formation [40]. This study showed that boron derivatives mainly control the function of QS system by increasing the expression of AI-2 synthetic protein LuxS in recombinant *E. coli.*

Figure 7c provides the effect of adding boron derivatives on the relative expression of recombinant *E. coli* flagellar movement genes (*motB, csgD,* and *fimC*). After adding H_3_BO_3,_ the expression of the *motB* gene was upregulated by 1.29-fold, the expression of *fimC* gene downregulated by 0.53-fold, and the expression of *csgD* was not significantly different from that in the control group. After adding NaBO_2,_ the expression of the *motB* gene upregulated by 1.31-fold, the expression of *fimC* gene downregulated by 0.58-fold, and the expression of *csgD* had no significant difference with the control group. Figure 7f illustrates the effect of H_3_BO_3_ concentration on flagellar movement genes. The expression level of *motB* was significantly upregulated at high concentrations from 0.8 mM to 1.0 mM H_3_BO_3_ treatment (*p* < 0.05). The expression level of *csgD* was significantly downregulated at high concentrations from 0.8 mM to 1.0 mM H_3_BO_3_ treatment (*p* < 0.05). The expression level of *fimC* was significantly downregulated at concentrations from 0.6 mM to 1.0 mM H_3_BO_3_ treatment (*p* < 0.05). *motB* was a subunit of flagellum [41], and upregulation of *motB* gene contributed to flagella and adhesion in *E. coli. csgD* is a fimbria synthesis regulatory factor, and *fimC* is a fimbrial adhesin [42]. Downregulation of *csgD* and *fimC* genes affected the motility of *E. coli* and contributes to the formation of biofilm. Thus, boron mediates the gene expression to regulate the biofilm formation as well as AI-2 and EPS concentrations in *E. coli*.

### 2.7. Boron Enhances Biofilm Formation to Affect Enzymatic Catalysis

As an active catalyst, biofilm can be widely used in the enzymatic catalysis of natural products. In this study, the recombinant *E. coli* can catalyze the conversion of mulberry flavonoid glycoside rutin to isoquercitrin. Boron can enhance biofilm formation and EPS concentration. It is unclear whether the addition of boron resulted in better catalytic ability in the recombinant *E. coli*. Figure S1a shows the effect of reusability of boron treated biofilm catalyst. With the addition of H_3_BO_3_, the yields of isoquercitrin of *E. coli* were higher than those in the control, especially in the second and third cycle. After three cycles, the biofilm catalytic yield of boron treatment decreased by 58.15%, while that of the control group decreased by 81.75%. Figure S1b shows the change of biofilm biomass during each cycle. It can be found that the boron treated biofilm biomass is higher than control group, especially in the first three cycles, which is basically consistent with the results of catalytic yield. In the first cycle, boron treatment increased biofilm biomass, but did not significantly improve catalytic yield, which may be due to the substrate rutin is not easy to diffuse in the thick biofilm [43], and some biofilms do not fully participate in the catalytic process. Therefore, boron derivatives can enhance the adhesion and synthesize biofilm in *E. coli* to stabilize the catalytic capacity of the intracellular enzymes.

## 3. Materials and Methods

### 3.1. Materials and Chemicals

The engineering bacteria used in this study was recombinant *E. coli* BL21 (DE3) which can secrete *α*-L-rhamnosidase [44]. Meanwhile, H_3_BO_3_, Na_2_B4O_7_, NaBO_2_, (NH_4_)_2_B_4_O_7_, NaBO_3_, B_2_O_3_, and K_2_B_4_O_7_, and the other reagents were analytical grade. The 24-well plate and the sterile glass slide were purchased from NEST Biotechnology Co., Ltd., (Wuxi, China).

### 3.2. Bacterial Strains and Culture Conditions

The recombinant *E. coli* was inoculated (1%, *v/v*) in LB liquid medium containing 50 μg/mL ampicillin and cultured at 37 °C at 220 rpm. *V. harveyi* BB170 was inoculated (1%, *v/v*) in marine broth 2216 medium The culture temperature was 30 °C, and the rotation speed was 220 rpm [23].

### 3.3. Effects of Different Boron Derivatives on the AI-2 Activity of Recombinant E. coli

The method of adding boron derivatives to the medium was carried out as previously described with slight optimization [17]. Different boron derivative (H_3_BO_3_, Na_2_B4O_7_, NaBO_2_, (NH_4_)_2_B_4_O_7_, NaBO_3_, B_2_O_3_, and K_2_B_4_O_7_) solutions were filter sterilized and added to LB medium, inoculated with 1% (*v/v*) recombinant *E. coli*, and cultured at 37 °C and 220 rpm. The concentration range of boron derivatives was 0.1–1.0 mM, with no boron derivative added to the control group. The bioassays of AI-2 activity in recombinant *E. coli* were optimized and performed based on Surette’s method [45]. *V. harveyi* BB170 was cultured overnight until the bacterial optical density (OD_600nm_) reached 0.8–1.2. Bacterial cultures were diluted with sterilized fresh AB medium at a ratio of 1:5000. The samples were mixed with diluted *V. harveyi* BB170 at a ratio of 1:50. Sterile supernatant of *V. harveyi* was selected as a positive control, and fresh sterilized AB medium was selected as a negative control. Bioluminescence was measured hourly using a multimode microtiter plate reader (Spectra Max i3, Sunnyvale, CA, USA).

### 3.4. Total Biomass Assay of Recombinant E. coli Biofilm

Boron derivatives were pre-added to LB medium, inoculated with 1% (*v/v*) recombinant *E. coli*, and cultured at 37 °C statically in 24-well plates. Crystal violet was used to assess the total biomass assay of recombinant *E. coli* biofilm with slight modification [46]. First, the excess medium was removed from the microplate and washed twice with PBS buffer (pH 7.4) after completing the biofilm culture. Second, the biofilms were fixed with 99% methanol for 15 min and then stained with 0.1% crystal violet for 30 min. Then, free crystal violet dye was washed away with PBS buffer and dried at room temperature for 30 min. Finally, the crystal violet bound to the biofilm was dissolved in 95% ethanol for 30 min. The total biomass assay of recombinant *E. coli* biofilm was measured spectrophometrically at an OD value of 595 nm.

### 3.5. Extraction of EPS

Five milliliters of recombinant *E. coli* culture medium was centrifuged at 4 °C and 10,000 rpm for 10 min to collect bacterial precipitates. Precipitates were rapidly suspended in NaCl solution (0.05%, 70 °C) to obtain a 5 mL mixture. Subsequently, the mixture was rapidly centrifuged and the supernatant was loosely bound EPS (LB-EPS). The bacterial precipitate deposition from LB-EPS was resuspended in 5 mL of 0.05% NaCl. The suspension was placed in a 70 °C water bath for 30 min, centrifuged, and the supernatant was tightly bound to EPS (TB-EPS). LB-EPS and TB-EPS were filtered through a 0.45 μm filter to remove impurities. The phenol–sulfuric acid method was used to quantify the content of polysaccharides, with glucose as the standard [47]. The protein content in EPS was determined according to the improved Lowry colorimetric method, and bovine albumin serum was used as the standard [48].

### 3.6. RNA Isolation and qRT-PCR

Recombinant *E. coli* treated with different boron derivatives were cultured overnight and cells were collected at the logarithmic growth phase. RNA was extracted by Trizol reagent (Sangon, Shanghai, China), and RNA integrity was evaluated by agarose gel electrophoresis. The cDNA was synthesized with a reagent kit (Takara, Dalian, China). Specific primers for qRT-PCR were designed and listed in Table S1. The *dnaE* gene was used as a reference for normalization. qRT-PCR was performed in triplicates using ChamQ SYBR qPCR Master Mix (Vazyme, Nanjing, China) with LightCycler^®^ 96 RealTime PCR System (Roche, USA) according to the manufacturer’s instructions. Quantification of expression levels of biofilm synthesis-related genes was performed using the 2^−ΔΔCt^ method [49].

### 3.7. Visualization of the Biofilms Using CLSM and SEM

Biofilms were grown on sterile glass slides (14 mm in diameter) in 24-well microtiter plates. Prior to scanning electron microscopy (SEM) and confocal laser scanning microscopy (CLSM) detection, the biofilm of recombinant *E. coli* was pretreated according to the method of Han et al. [50]. First, the biofilm was incubated at 37 °C for 12 h in 24-well microtiter plates, and were gently rinsed three times with PBS buffer to remove the suspended bacteria. Second, the biofilms were fixed on glass slides with 4% glutaraldehyde (Sangon, Shanghai, China) at 4 °C overnight. Subsequently, the biofilms were gently rinsed three times with PBS buffer to remove the glutaraldehyde. The biofilms were fluorescently stained for 30 min with SYBR Green Ⅰ (Sangon, Shanghai, China), then gently rinsed three times with PBS buffer to remove the SYBR Green Ⅰ. Finally, sterile glass was taken out and dried for 20 min. Diagrams were captured by the SEM (Regulus-8100, Hitachi, Japan) and CLMS machine (LEXTOLS4000, Olympus, Japan).

### 3.8. Statistical Analysis

All research subjects and parameters were subjected to three repeated experiments. The stability of the data was tested by the standard deviation, the statistical data were processed by Excel, and the data (x ± s) were subjected to a one-way analysis of variance (ANOVA).

## 4. Conclusions

In this study, the stress response of biofilm formation to boron derivatives was illuminated by determining the quorum sensing (QS) system autoinducer-2 (AI-2) activity, extracellular polymeric substances (EPS) composition analysis, linear correlation, analysis of gene expression levels, etc. The results show that the addition of boron derivatives can regulate the activity of AI-2 in the QS system, accelerate the synthesis of EPS, and promote the adsorption and growth of recombinant *E. coli* biofilm. AI-2 activity had a good linear correlation with LB-EPS and TB-EPS at low concentrations of boron derivatives. Boron derivatives can significantly upregulate the transcription levels of biofilm synthesis genes. Additionally, boron promoted biofilm formation by upregulating the expression levels of biofilm-related genes, improving the QS system AI-2 activity, and increasing EPS secretion. Meanwhile, H_3_BO_3_ can enhance the biofilm formation to mediate the recombinant strain and stabilize the catalytic ability of intracellular enzymes. The strengthening effect of boron on recombinant *E. coli* biofilm will provide a novel insight to unlock the efficient control of gram-negative and gram-positive bacteria in biofilm catalysis.

## Figures and Tables

**Table 1:** The average thickness of the biofilms in the corresponding CLSM diagrams.

Treatment Group	Sp (μm)	Sv (μm)	Sz (μm)	Average Thickness (μm)
Control	7.16	5.84	13.00	4.80 ± 0.78 ^c^
Na_2_B_4_O_7_	8.61	9.30	17.91	5.63 ± 0.28 ^c^
NaBO_3_	7.49	8.15	15.64	5.66 ± 0.75 ^c^
NaBO_2_	10.23	13.59	23.82	8.11 ± 0.85 ^b^
H_3_BO_3_	14.06	15.86	29.91	11.13 ± 1.17 ^a^

Sp is the maximum peak, Sv is the maximum valley, and Sz is the maximum thickness of the biofilm. The different letters above the bars indicate significant differences (*p* < 0.05).

## Supplementary Materials

The following supporting information can be downloaded at: https://www.mdpi.com/article/10.3390/ijms23158059/s1.
