# Supplementary material for: Boron Derivatives Accelerate Biofilm Formation of Recombinant Escherichia coli via Increasing Quorum Sensing System Autoinducer-2 Activity
Source: Int J Mol Sci. 2022 Jul 22;23(15):8059. doi: 10.3390/ijms23158059 (PMC9332218; doi:10.3390/ijms23158059)
Supplement: Supplementary file 1 [file ijms-23-08059-s001.zip › ijms-1823888-supplementary.pdf]

Supplementary materials for

Boron derivatives accelerate biofilm formation of recombinant *Escherichia coli via* increasing quorum sensing system autoinducer-2 activity

Huan Chen [1], Cheng-Hai Yan [1], Yu-Fan Zhan [1], Li-Tian Geng [1], Lin-Lin Zhu [1], Lu-Chan Gong [1, 2], Jun Wang [1, 2, *]

[1] Jiangsu Key Laboratory of Sericultural Biology and Biotechnology, School of Biotechnology, Jiangsu University of Science and Technology, Zhenjiang, Jiangsu 212100, P R China

[2] Key Laboratory of Silkworm and Mulberry Genetic Improvement, Ministry of Agriculture and Rural Affairs, Sericultural Research Institute, Chinese Academy of Agricultural Sciences, Zhenjiang, Jiangsu 212100, P R China

* Correspondence: wangjun@just.edu.cn; Tel.: +86-511-84448290

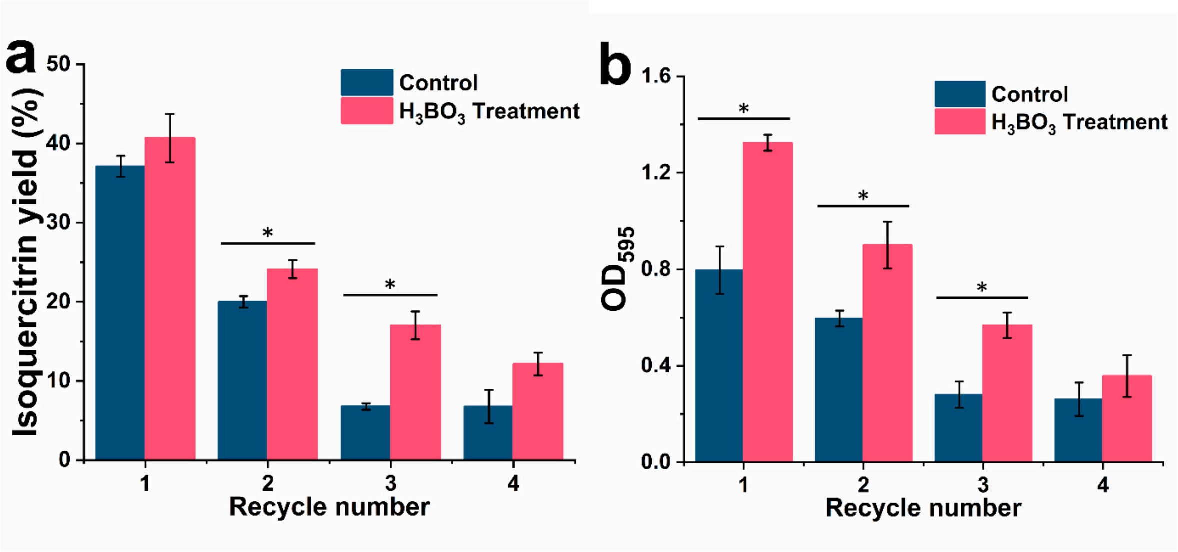

Figure S1 Reusability of boron treated biofilm catalyst on the yield of isoquercitrin (a) and biofilm biomass (b). Reaction conditions: substrate concentration 0.05 g/L; temperature 40℃, each reaction cycle 2.5 h. *$p < 0.05$: significant compared with the control.

Table S1. Primers used for qRT-PCR.

Primers	Sequence (5' to 3')	bp	GenBank ccession No.
rfaP-F	GCGGCACGACCCTGAAAGAGAT	171	NP_312532.2
rfaP-R	ACGAGGTTCTGGTCAGCGGATT		
wza-F	TGACGGATACAGCCAGCGACAT	148	NP_310894.1
wza-R	TCCAGCGGACTCCACTTTCAGT		
pgaA-F	ACGCACCAGGAAATCAGGGACT	178	NP_415543.1
pgaA-R	GCTGCCTGCTGCCATTCTTGT		
bcsA-F	ACGCCGCACCACTTCTTCTCA	157	NP_417990.4
bcsA-R	GACGAATCACCGCACAGGAACC		
luxS-F	GCTGCTCATCTGGCGTACCAAT	175	NP_417172.1
luxS-R	TGCGTGCCGAACAAAGAAGTGA		
lsrB-F	GTGAGAGTGCTGACCTGGGACT	108	NP_310150.1
lsrB-R	CGCCGCCATATCCACCAACAT		
aphA-F	CGCTCGTGGTATCCGCATTCTG	118	NP_418479.1
aphA-R	CGCTCTCCCGCTCTGTCAGTAT		
motB-F	TCCACTGGCGACTGCGGTTA	125	NP_416403.1
motB-R	GCTCTTCGATGTTCGGCTGCTT		
csgD-F	ACAGCACGGAATCAGTCCTCCT	186	NP_415558.1
csgD-R	TTGCCCAGGAAACCGCTTGTG		
fimC-F	ACGCTGATTAACCCGACACCCT	106	NP_418736.3
fimC-R	TAACCGTGCTTTCGCCCATTGG		
dnaE-F	GCGTTATGTCGGAGGCGTAAGG	144	WP_012815364.1
dnaE-R	CGGTCATCCAGCGTGCAGATAC		
